# Nutrition Society of New Zealand Annual Conference Held in Christchurch, New Zealand, 8–9 December 2016

**DOI:** 10.3390/nu9040348

**Published:** 2017-04-01

**Authors:** Rachel Brown, Sheila Skeaff

**Affiliations:** Department of Human Nutrition, University of Otago, Dunedin, 9054, New Zealand; sheila.skeaff@otago.ac.nz

## 1. Preface

The annual conference and scientific meeting of the Nutrition Society of New Zealand took place in Christchurch, New Zealand from 8–9 December 2016. The meeting comprised 2 plenary sessions, 2 concurrent oral sessions, and 17 posters, providing an opportunity for more than 43 nutritional scientists to present their research. Abstracts for plenary talks, oral presentations and posters are published here. The aim of the annual meeting is to foster discussion and disseminate the results of nutrition-related research undertaken by the members of the society.

The theme of the Nutrition Society of New Zealand in 2016 was “Feeding our Society: Manaakitanga in Nutrition for Aotearoa (i.e., New Zealand)”. Maanatikanga is a Māori word that refers to hospitality, kindness, generosity, and support. The first plenary session included presentations on maanatikanga in nutrition by Leonie Matoe, food loss and food security by Dr Ian Fergusson, food costs by Dr Louise Mainvil, food insecurity by Associate Professor Winsome Parnell, and the role of poverty in nutrition in Aotearoa/New Zealand by Christina McKerchar. The second plenary session included presentations on food writing by Niki Bezzzant, meeting the diverse nutritional needs of older adults by Dr Carol Wham, early life approaches to obesity prevention by Associate Professor Rachael Taylor, nutrition and nutrients to treat mental illness by Professor Julia Rucklidge, and ‘food talks better than we can’ by Bronwen King. The Muriel Bell Lecture entitled ‘Evolution not Revolution: Nutrition and Obesity’ was given by Professor Elaine Rush, Auckland University of Technology. 

## 2. Summary of Scientific Presentations

### 2.1. Evaluating the Effects of a Food Assistance Programme on the Dietary Intake of Year 5 and 6 Students from a Low-Decile School in South Auckland

Ansell, S., Walia, N., Breier, B.H. and Wham, C.

Background: The food assistance programme (FAP) is conducted by a charitable organisation that provides lunch on alternate week-days, to low-decile schools during winter. The aim of this study was to evaluate the effects of the FAP on dietary intake during school hours.

Methods: Supervised self-administered weekly food records were completed by 82 year five and six students from a low-decile school when the FAP was in operation (FAP week) and when the FAP was not in operation (control week). Mean dietary intake of energy and key nutrients were compared with estimated school day requirements (40% of the Nutrient Reference Values).

Results: During FAP week, compared to the control week, students consumed more energy, protein, carbohydrate, fat, saturated fat and dietary fibre (*p <* 0.050). Estimated school-day dietary fibre requirements were only met by girls (9.13 ± 7.39 g/day). As % energy, protein increased in boys from 11.5% to 13.6% (*p =* 0.006). However, protein as % energy did not meet recommendations in either boys or girls. Overall during both FAP and the control week consumption of saturated fat exceeded the estimated 40% of NRV requirements for both boys and girls (*p <* 0.050).

Conclusions: In comparison to the control week, the FAP provided a better quality source of energy and macronutrients to students, including more dietary fibre. However, energy density of the FAP should be moderated, in particular the saturated fat content of meals served. A well-designed FAP for low-decile schools can have a beneficial impact on dietary intake of children. Furthermore, the ingredients used strongly influence the nutritional quality of meals provided.

### 2.2. Totara House Healthy Eating Study. The Feasibility of Adapting the Senior Chef Programme for Patients in a Mental Health Setting

Borich, A., Elmslie, J.

Background: People experiencing early or first episode psychosis are at increased risk of metabolic complications resulting from medication side-effects and lifestyle choices. Few studies have explored practical nutrition education strategies to improve health outcomes in this population. This study aimed to assess whether the Senior Chef programme could be adapted to meet the needs of the patients at Totara House, a CDHB outpatient service for young adults with early or first episode psychosis.

Methods: Participants (staff, patients and family members/carers) were recruited by advertisement or through recommendation from Totara House staff. Their opinions on the importance of nutrition what content should be included and communication strategies, were explored by means of a focus group or individual interview. Participants completed a questionnaire to assess their level of nutrition knowledge.

Results: The data were analysed using thematic analysis. Four main themes emerged: personal values, knowledge and understanding of health, previous experiences with physical activity/nutrition, and specific recommendations for the programme. These themes support a programme with a practical component, social atmosphere and recipes/information applicable to their situation. The questionnaire scores showed an average level of nutrition knowledge, mean score 53% (*n =* 24). No significant difference was observed when analysed by group (patients 49% (*n =* 8), staff 55% (*n =* 10), family/carers 56% (*n =* 6)), range 32%–73%. 

Conclusion: A practical nutrition education programme based on Senior Chef would be valued by Totara House staff, patients and families. To ensure that the content adheres to current evidence-based nutrition advice, a registered dietitian should be involved in running or overseeing the programme.

### 2.3. Developing a Methodology to Assess the Cost and Affordability of a Healthy Diet versus the Current Diet for Pacific People in New Zealand

Buch, T., Mackay, S., Beck, K.L., Swinburn, B. and Funaki-Tahifote, M.

Background: Price and affordability of diets are important determinants of food choice, and diet quality has been linked to socioeconomic factors. The issue of diet cost becomes particularly important for Pacific people in New Zealand due to health inequalities.

Methods: A 14-day ‘healthy diet’ meeting food and nutrition guidelines, nutrient reference values, and energy requirements for a healthy BMI, and a ‘current unhealthy diet’ based on eating patterns identified from national nutrition surveys and energy requirements for current BMI were developed for Pacific people. Two expert panels were conducted with Pacific people knowledgeable in food and dietary patterns of Pacific communities to ensure culturally appropriate foods were included and to provide feedback on results. Food prices were collected from three supermarkets, three fruit and vegetable shops and three bakeries in South Auckland. Cost and affordability of the diets was assessed for a household of six. Only the ‘current’ diet contained alcohol, takeaways or discretionary foods. A range of cost scenarios were calculated by adding back equivalent alcohol, takeaways and energy to the ‘healthy’ diet.

Results: The ‘healthy’ and ‘current’ base diets were similar in cost ($395/$413), but when including equivalent alcohol, takeaways or energy there were differences depending on the criteria used. If households were on the minimum wage, the ‘healthy’ and ‘current’ diets required 26% and 27% of household income respectively. Feedback from the expert panel was positive with an interest in using the results as a teaching tool.

Conclusion: The relative price and affordability of a ‘healthy’ and ‘current’ diet for Pacific people in New Zealand was similar, however altering some aspects of the diet changed the cost difference. It is essential when assessing diet cost for cultural subgroups within a population to consult with these groups in order to better understand commonly consumed foods and dietary patterns.

### 2.4. The Breast Milk Composition of Different Ethnicities of Women Living in New Zealand

Butts, C., Paturi, G., Hedderley, D., Sims, I., Bell, T., Martell, S., Dinnan, H. and Herath, T.

Background: Human milk is rich in nutrients, complex in its composition, and has a far-reaching effect on a baby’s health through its role in nutrition, gastrointestinal tract development, and immune health. 

Methods: A pilot observational study was undertaken to compare the breast milk composition and diet records from mothers from different ethnic groups within New Zealand (NZ). Eighty participants were enrolled from the Manawatu-Whanganui region. The participants completed a three-day diet record immediately prior to collecting three breast milk samples over a one-week period between 6 and 8 weeks postpartum. 

Results: We obtained information and samples from 8 Asian, 15 Māori, 2 Pacific Island and 53 NZ European mothers (total recruited 78). The proportions of the different ethnicities participating in the study were 68% NZ Europeans, 19% Māori, 10% Asian and 3% Pacific Island. There were significant differences (*p* < 0.05) in nutrient intakes and breast milk compositions. Asian women consumed more folate, vitamin A, iodine, and unsaturated fats and less fat than the other ethnic groups, and their breast milk contained higher (*p* < 0.05) amounts of polyunsaturated fatty acids, omega-3 and 6 fatty acids, docosahexaenoic and linoleic acids. Spearman rank correlations showed negative effects of saturated fat intake on breast milk concentrations of polyunsaturated, omega-3, omega-6, linolenic, and linoleic fatty acids.

Conclusion: The ethnic representation of mothers in this study was similar to that found in the New Zealand population. The study has shown that the diets of New Zealand mothers influence their breast milk composition and therefore the nutrients their babies receive.

### 2.5. A Snapshot of Milk Types Consumed by New Zealand Preschool Children

Cairncross, C.T., Grant, C.C., Stonehouse, W., Conlon, C.A., McDonald, B., Houghton, L.A., Camargo Jr., C.A., Coad, J. and von Hurst, P.R.

Background: As a nutrient dense food, milk is generally considered a healthy food for children. Currently, the Ministry of Health recommends parents gradually introduce reduced fat milks to children from age two. The aim was to investigate the types of milk consumed by NZ preschool children. 

Methods: We recruited 1329 preschool children (2 to <5 years), throughout NZ. Parents were asked to record all types of milk their child consumed. Demographic data were collected and descriptive statistical methods used. 

Results: Standard (whole) milk was the most commonly consumed milk type (64%), followed by reduced fat (22%) and trim (7%). Consumption of full-fat milk was highest in children at age 2, of Pacific and Maori ethnicity and of lower socio-economic status. Parents reported that 6% of children did not drink milk, while a further 6% consumed toddler milk formulations.

Conclusion: Only a small number of children met the NZ guideline, which is recommended to reduce saturated fat intake from this major dietary source. Previously reported barriers to reduced-fat milk consumption include perception of inferior taste and higher cost compared to standard milks, which was reflected in the lower socio-economic status of the children who were reported to drink standard milk. There may be nutritional implications for those reported not to drink milk, affecting future health outcomes. A high proportion of the children consumed full-fat milk despite current NZ recommendations. Addressing barriers to consumption of reduced-fat milks in this age group would require targeted public health initiatives. 

### 2.6. Prediction Equations Overestimate the Energy Requirements of Obesity Susceptible Individuals

Cooke, R., Taylor, R., Skidmore, P., Jones, L. and Brown, R.

Background: An evaluation of energy requirements is a necessary part of dietary counseling, particularly when weight reduction is the goal. In many instances rather than actually measuring resting metabolic rate (RMR), predictive equations are used to estimate the RMR of individuals in a clinical setting. Our objective was to compare the measured and predicted RMR of individuals who remain lean despite living in an obesogenic environment (obesity resistant individuals) with those who struggle to maintain a healthy body weight and report having to consume smaller amounts of food (obesity susceptible individuals).

Methods: Obesity resistant individuals (ORI) and obesity susceptible individuals (OSI) were identified using a simple 6-item screening tool. Measurement of RMR was undertaken in 31 ORI (14 females, 17 males) and 26 OSI (14 females, 12 males) 12 h after an overnight fast, using indirect calorimetry and following standard procedures. Predicted RMR was calculated using the FAO/WHO/UNU (Food and Agricultural Organisation/World Health Organisation/United Nations University), Oxford and Miflin-St Jeor equations and compared to measured RMR.

Results: Absolute RMR was significantly lower in ORI versus OSI (748 kJ·day^−1^, 95% CI: 52, 1443; *p =* 0.036); however, relative RMR was significantly lower in OSI compared to ORI (−15 kJ·kg·BM^−1^·day^−1^, 95% CI: −24, −6; *p =* 0.001) and lower in female OSI compared to all other groups (all *p* ≤ 0.001). The RMR of OSI and ORI females differed by 25.2 kJ·kg^−1^·day^−1^. Given the mean weight of OSI females was 85.5 kg this equates to a difference of 2155 kJ·day^−1^. All three prediction equations over-estimated RMR to some extent in both ORI and OSI but this difference was significant for OSI females (1664, 1466 and 1422 kJ·day^−1^, FAO/WHO/UNU, Oxford and Miflin-St Jeor equations respectively).

Conclusions: The use of prediction equations may lead to an overestimation of RMR and subsequently energy requirements particularly in females who identify as being susceptible to obesity.

### 2.7. Utilisation of Fish as a Protein Supplement to Enrich Snack Foods for Better Nutrition 

Desai, A.S., Brennan, C.S. and Brennan, M.A.

Background: Starch-based snack foods from corn, rice and wheat are rich in carbohydrate and low in protein. The composition and processing of these snack foods means that they possess a high digestible starch content which in turn raises nutritional and health issues. Limited work has been carried out on the enrichment of snack products with fish protein. Therefore, there is a need to conduct research on the utilisation of fish and fish waste for the enhancement of snack food quality.

Results: Fish proteins are rich in all the essential amino acids (particularly methionine, lysine, histidine, threonine and valine). This contrasts to most protein from plant sources such as wheat, rice, maize, barley, soybean and pea, which lack adequate amounts of one or more essential amino acids. A limited supply of lysine leads to mental and physical disability as the lysine is the main precursor for the synthesis of glutamate, which is a significant neurotransmitter in the mammalian nervous system. Protein utilisation of food products should increase due to a well-balanced amino acid composition in fish. Fish also contains omega-3 (*n*-3) fatty acids including eicosapentaenoic acid (EPA) and docosahexaenoic acid (DHA). These fatty acids are supplied solely by the diet as they cannot be synthesised by the human body. They reduce risk factors associated with cardiovascular disease, hypertension, inflammation, asthma, arthritis, psoriasis and cancer. Antioxidant peptides will be obtained from the fish material with the use of enzyme hydrolysis and characterised by their amino acid sequence and composition. These peptides can be used as natural antioxidants in the lipid oxidation of food products with no toxic effect. 

Conclusions: Enrichment of snack products with fish material should significantly improve the nutrient profile and help fulfil the requirement for essential amino acids and fatty acids. Also, the high protein content could lead to a decrease in the predicted glycaemic impact. 

### 2.8. Food Loss, Food Security, and Should We Be Worried?

Ferguson, I.B.

Food security means many things to different economies, industries, public bodies and individuals. Security means nutritional sustenance, sufficiency and maintenance of supply, integrity of the supply and value chain, and continued and improved trade access. Because of all these aspects, food security is high on the agendas of individual jurisdictions, and international political and economic bodies such as APEC and FAO. This is further accentuated by predictions of a world population of ~9 billion by 2050 and the negative consequences of climate change on food production. 

Ensuring security of supply and nutritional value does not, however, only demand adaptive production systems and increased productivity, but also attention to the whole supply chain. For instance, tackling substantial food waste and food loss is a core requirement in food production systems; over 30% of fresh food may commonly be lost across the supply chain, both re- and postharvest. The security of food production and nutritional value for future populations means that we must address the science and economics of all steps of the chain, from genetics to the consumer. New Zealand has much to offer—while it has been proposed that we can only feed about 40 million people, our knowledge and adaptable systems can show others how to increase and secure high value and high nutrition food supplies in a rapidly changing global environment. 

### 2.9. Using the Behavioural Paediatric Feeding Assessment Scale to Identify Fussy Eaters, and Their Adherence to Dietary Guidelines

Hashimoto, S., Von Hurst, P.R. and Conlon, C.A.

Background: Childhood feeding issues range from problems with few immediate health risks to significant problems requiring medical intervention. Fussy eating is implicated in low critical nutrient intake and poor eating habits, increasing risk of later chronic disease. A simple tool to assess fussy eating is not available. This study aims to determine if the Behavioural Paediatric Feeding Assessment Scale (BPFAS) can identify child fussy eaters and assess risk of poor adherence to dietary guidelines. 

Methods: Parents of 1389 New Zealand children aged 2–4 years were recruited through online- and print-media to complete an online questionnaire. Data were collected on: incidence of problem mealtime behaviours using the Total Frequency Score (TFS) from BPFAS; adherence to Ministry of Health (MoH) dietary guidelines using the Dietary Index for a Child's Eating (DICE) and parental perceptions of fussiness. 

Results: Scores ≥ 81 on the TFS were stratified into the clinical feeding problem group (22.7%). TFS for normative and problem groups were 62.6 ± 9.98 and 92.4 ± 10.5 respectively. The problem group had poorer DICE scores (81.9 ± 12.3) than normative group (91.8 ± 9.23). There were overall moderate inverse correlations (*r* = −0.45, *p <* 0.001) between DICE and TFS, and between DICE and parentally-perceived fussiness score (*r* = −0.42, *p <* 0.001). A strong positive correlation between TFS and parentally-perceived fussiness score (*r* = 0.72, *p <* 0.001) was also found in the total group. These relationships remained significant when analysis was repeated only on the normative group. 

Conclusions: These results indicate that normative eaters who display more problem mealtime behaviours adhere less to MoH guidelines than those with fewer problem mealtime behaviours. Children who have more problematic mealtime behaviours are also classed as fussy eaters by their parents, suggesting the BPFAS can be used to identify fussy eaters. 

### 2.10. SouthSci—Participatory Science Platform, South Auckland Pilot: Supporting a Teacher- and Student-Led School Nutrition Project to Showcase the Value of Nutrition Science Skills to South Auckland students

Higginson, C.A., Stewart, N. and Morgan, S.

Background: SouthSci is the South Auckland pilot of the Participatory Science Platform funded by the New Zealand Government, encouraging community groups to develop scientific projects that address issues of concern to them, and to apply for financial and professional scientific assistance to support their investigation. One example is Conifer Grove School’s Year 7 nutrition project, investigating the prevalence of processed and pre-packaged foods within the school and wider school community (whānau), and its potential implications on student behaviour, academic achievement and general health.

Methods: Students compared differences between everyday foods and their various ingredients, and effects on parameters such as shelf-life and nutritional value. A student-created questionnaire assessed knowledge and attitudes pertaining to pre-packaged foods and their ingredients and the wider school community (whānau). Expertise of Food Technologists and Nutritional Scientists was utilised to bolster in-class learning and enhance students’ understanding of the industrial and corporate basis of pre-packaged food production.

Results: Under robust teacher leadership, students successfully synergised Level 4 Science, Technology, Mathematics and English curricula with a real world nutritional issue of importance to them, and the wider population. This was achieved through engagement in experimental science, preparation of a knowledge and needs assessment questionnaire, data collection and associated statistical analyses, and a student visit to Fonterra’s Tip Top factory. This enabled students to experience commerical-scale food production through a New Zealand company that has removed articifical colours and flavours from their products.

Conclusion: Enthusiastic engagement from students during Conifer Grove School’s SouthSci project exemplifies the importance of student-led, nutrition-specific scientific inquiry in the wider understanding of the New Zealand, and global, nutrition environment, and its implications on health and wellbeing. The program has built rapport between students and the professional scientific world. It is an exciting and emerging means of engineering a nutritonally conscious generation, and incorporates scientific inquiry into everyday life experiences.

### 2.11. Body-Composition Assessment Using Air Displacement Plethysmography in Healthy Term Infants

Ichhpuniani, B., von Hurst, P.R., Mugridge, O. and Conlon, C.

Background: Ethnic differences in fat mass (FM) have been reported in adults and children but it is currently unknown whether these differences are evident shortly after birth. Significant differences in birth weight, which comprises FM and fat free mass (FFM), have been observed in different ethnic groups in New Zealand (NZ). The aim of this study was to examine the differences in FM and FFM using air displacement plethysmography (ADP) between NZ European (reference group), Māori, Pacific, Asian and South Asian healthy term infants.

Methods: Body composition, including FM and FFM, weight, length, head circumference and waist circumference, was measured in a convenience sample of healthy term infants (*n =* 255) shortly after birth. Ethnicity was classified using standard ethnicity data protocols. Statistical analysis included independent t-tests and multiple linear regression analysis.

Results: Body composition was assessed at a mean age of 1.7 ± 0.85 days, while adjusting for gender and postnatal age. South Asian infants had significantly lower FFM (2691.7 ± 389.7 g vs. 2938.6 ± 364.0 g, *p =* 0.006) and weight than NZ European infants (3045.5 ± 535.2 g vs. 3352.3 ± 575.8 g, *p =* 0.014). They also had the smallest head (34.2 ± 1.7 cm vs. 35.4 ± 1.7 cm, *p =* 0.002) and waist circumference (31.5 ± 3.0 cm vs. 33.2 ± 2.1 cm, *p =* 0.003). Waist circumference of Asian infants was also significantly smaller than NZ European infants (32.3 ± 2.1 cm vs. 33.2 ± 2.1 cm, *p =* 0.044). When categorised by gender, males had significantly greater FFM, weight, length and head circumference (*p <* 0.05). No gender or ethnic difference was noted in FM (g) or %FM.

Conclusions: This is the first study in New Zealand to report body composition in healthy term infants using ADP and the findings justify further prospective investigation in a larger sample size to investigate the significance of the differences identified. 

### 2.12. The Impact of Promoting Healthier Drink Options within a Quick Service Restaurant Setting

Kenny, S., Greig, F. and Schramm, C.

Background: There has been much discussion on the role of quick service restaurants in population health. As part of McDonald’s NZ’s ongoing commitment to improving menu nutrition and choice, a trial was undertaken to identify mechanisms that encourage customer behavior change towards healthier drink options. 

Methods: McDonald’s undertook a three-cell trial from July 2014–January 2015, across 37 restaurants nationwide. Restaurants were allocated one of three sales interventions; 1. suggestive selling by crew for Coke Zero, 2. suggestive selling by crew for Coke Zero, display of only Zero/Diet drink options on menu boards, and suggestive selling of water in Happy Meals. 3. suggestive selling by crew for Coke Zero, display of only Zero/Diet drink options on menu boards, and an option to switch the drink in combos to 600 mL Kiwi Blue water. Results were analysed via a point of sale system. 

Results: Approximately 9 million customers visited the restaurants during the trial. There were no statistically significant changes in behavior for interventions 1 or 2. Intervention 2 obtained the highest conversion to Coke Zero. Intervention 3 achieved a significant increase in water purchasing behavior with a 41.9% increase in Kiwi Blue units.

Conclusions: The trial demonstrated customers were more likely to opt for healthier drink options if visually prompted at point of purchase and healthier alternatives were available at an equivalent price. As a result of the trial, a revised version of intervention 3 has been implemented throughout McDonald’s NZ restaurants, including the addition of an a la carte 600 mL water for $2. 

### 2.13. Fat Sensation: Are Fat Taste and Olfaction Sensitivity Linked with Eating Behaviour?

Kindleysides, S., Beck, K.L., Walsh, D.C.I., Henderson, L., Jayasinghe, S.N., Golding, M. and Breier, B.H.

Background: Perception of fat taste, fat aroma and fat texture are hypothesised to influence food preferences, thus shaping dietary intake and eating behaviour and consequently long-term health. We therefore characterised taste and olfactory threshold measurements of oleic acid (C18:1) and investigated associations between fat taste, olfaction, mouthfeel of fat, and dietary intake, eating behaviour and anthropometric measurements. 

Methods: Fifty participants attended three sessions to assess oleic acid taste and olfaction thresholds. Height and weight, the olfactory threshold for *n*-butanol and subjective mouthfeel ratings of vanilla custard samples were also assessed. Dietary intake and eating behaviour were evaluated using a Food Frequency and a Three-Factor Eating Questionnaire, respectively. 

Results: Binomial regression analysis was used to model fat taste and olfaction data, and revealed the ability to perceive fat varied immensely across participants. Taste and olfactory detection for oleic acid were significantly correlated (*p <* 0.02). Oleic acid taste hypersensitivity was associated significantly with *n*-butanol aroma sensitivity (*p <* 0.03), and was associated with discrimination of fat content in custard (*p <* 0.03) and with lower body mass index (*p <* 0.05). Analyses of eating behaviour showed that disinhibition was higher in women who were hyposensitive to fat taste (*p <* 0.05). Servings per day of legumes, nuts and seeds was significantly correlated with high olfactory sensitivity to oleic acid (*p <* 0.01).

Conclusions: These findings suggest a link between fat taste and olfaction sensitivity. Furthermore, fat taste is associated with eating behaviour. A better understanding of the relationship between fat taste perception and our diet will lead to new strategies that reduce chronic disease risk.

### 2.14. Food Talks—Better than We Can!

King, B.

With increasing interest in Nutrition, as a science, as a medical intervention and in the media, instead of being better informed, the average person is more confused than ever. Even health professionals can’t seem to agree on what is the optimum diet for health and the solutions to the burgeoning obesity epidemic have us baffled!

When arguing over specifics gets in the way of action, it’s time to take stock and focus on what we know works. And motivating people through the taste of healthy food is a simple and effective strategy—food is a language people understand, food talks better than we can!

This is the basis of 2 lifestyle programmes I manage—Appetite for Life, a healthy lifestyle and weight management programme delivered via Pegasus Health in Primary Care, and a Nutritionist in Supermarkets Programme delivered in South Island New World Supermarkets.

### 2.15. Quality Characteristics and Sensory Evaluation of Tomatoes (Solanum lycopersicum L.) Grown under Drought Stress in a Greenhouse 

Klunklin, W., Hofmann, R.W. and Savage, G.P.

Background: Tomatoes are one of the most nutritionally and economically important crops around the world and in New Zealand. Tomatoes require a lot of water to stay alive and face a severe drought stress. However, few studies have evaluated the physicochemical characteristics and sensory evaluation of commercial tomatoes grown under water stress conditions. In this experiment tomatoes grown in a greenhouse under water-deficit conditions were compared to well-watered tomatoes. The physicochemical properties, antioxidant contents and sensory evaluation of the fruits were compared.

Methods: Four tomato cultivars (Incas, Marmande, Scoresby Dwarf and Window Box Red) were grown under well-watered and drought stress conditions in a greenhouse and the tomatoes were harvested when ripe.

Results: There were significant differences between cultivars in the quality characteristics such as dry matter, total soluble solids, pH and colour parameters but there was no difference between the two treatments in the fruits (*p* > 0.05). However, there was a significant difference in the antioxidant content between two treatments of the fruits. Analysis of a triangle taste test showed that a significant difference (*p* < 0.05) between Incas and Marmande tomatoes grown under well-watered and drought stress conditions could be observed but the panellists were unable to taste the differences between the other two cultivars. 

Conclusions: No effect on the quality characteristics or colour parameters could be observed between the watered and the drought treated fruits. Interestingly, the panellists could detect a difference in the taste of the tomatoes grown under the two different treatments for only two cultivars (Incas and Marmande). 

### 2.16. Exploring the Dietary Intake of New Zealand European Women with Different Body Composition Profiles

Kruger, R., Schrijvers, J.K., Beck, K.L. and McNaughton, S.A.

Background: Dietary intake contributes significantly to body composition; body fat content may vary as a result in women within the same BMI category. The aim was to investigate dietary intakes of young New Zealand European (NZE) women, and within different body composition profiles (BCP).

Methods: Post-menarche, pre-menopausal NZE women (16–45 years) completed a validated 220-item, self-administrated, semi-quantitative food frequency questionnaire (FFQ) assessing dietary intake over the previous month. Body mass index (BMI, kg/m^2^) was calculated from height and weight; body fat percentage (BF%) was measured using air displacement plethysmography (BodPod). Participants were categorised into one of three BCPs: normal BMI (18.5–24.9 kg/m^2^), normal BF% (<30%) (NN); normal BMI, high BF% (≥30%) (NH); high BMI (≥25 kg/m^2^), high BF% (HH). Food and nutrient intakes were examined. 

Results: Insufficient intakes below the Estimated Average Requirements (EAR) were observed for Vitamin D (64%), Energy (23%), Folate (25%), Selenium (23%), Calcium (17%), Magnesium (8.2%), Thiamin (7.7%), Vitamin B12 (5%), and Iron (6%). Percentage of energy intake was outside the acceptable macronutrient distribution range (AMDR) for carbohydrate (below the AMDR, mean ± SD 41.9% ± 7%) and saturated fat (above the AMDR, 13.9% ± 3.5%). Fewer serves of fruit and vegetables and more of diet soft drinks, chocolate bars and cooking oil were consumed by the HH BCP; they also had lowest calcium (1159.5 mg/day) and highest energy (9296 kJ/day), total (89.4 g/day) and saturated (36.5 g/day) fat intakes. No significant associations were found with BF%. Vitamins A, E, D, and zinc intakes were adequate, and comparable between BCPs.

Conclusions: Pre-menopausal NZE women are at risk of nutritional deficiencies due to poor intakes, irrespective of body fatness. NZE women consume diets low in carbohydrates and high in saturated fat which deviates from the current dietary guidelines, and should therefore be addressed in efforts to improve women’s dietary quality. 

### 2.17. The Effect of a Nutrient Supplement on Vitamin D Serum Concentrations in Healthy People

Laing, B.B., Aggio, R., Ellett, S., Marlow, G., Jesuthasan, A., Lock, D., Eyres, L. and Ferguson, L.R.

Background: Vitamin D_3_ is associated with immune regulatory functions. Vitamin D deficiency has also been associated with increased risk of diseases in adults such as osteomalacia, inflammatory bowel disease, cancer, diabetes, hypertension, heart disease and multiple sclerosis.

Methods: In Auckland, New Zealand, a randomised, controlled, double blind, cross-over intervention 12 week trial on healthy people was conducted to measure the effect of a nutrient supplement which contains vitamin D_3_ and long-chain Omega 3 fatty acids. The control used was medium chain triglyceride oil (i.e., MCT-8 + 10 Triglyceride). The supplement was trialled to ascertain its effects on the serum concentrations of 25(OH)D_3_. The levels of C-reactive protein (CRP) and lipid peroxidation in plasma and compliance were measured at baseline and at the end of each phase. The effects of vitamin D3 supplementation was assessed by a general linear mixed model (GLMM) and ANOVA. 

Results: The ANOVA analysis showed a significant effect of treatment and no period effect. For those taking the nutrient supplement first, the GLMM analysis showed a significant increase in serum concentration of 25(OH) D_3_ (*p <* 0.001) and a significant decrease in CRP (*p =* 0.011). For those participants receiving placebo first, the GLMM analysis indicated a significant decrease in serum concentrations of 25(OH)D_3_. There was no evidence of lipid peroxidation or non-compliance in biomarker measures. 

Conclusions: The results reported here indicate that the nutrient supplement tested was effective in increasing serum concentrations of 25(OH)D_3_. However, the placebo used appeared not to be neutral in its effect, as it significantly lowered serum concentrations of 25(OH)D_3_.

### 2.18. Cereal Bran Inclusion in Chinese Steamed Bread: Potential Reduction in Glycaemic Response and Effects on Dough Quality

Liu, W., Brennan, C., Brennan, M. and Serventi, L.

Background: Dietary fibre (DF) has many beneficial physiological effects, such as reduction of blood cholesterol levels, and reduction in glycaemic impact (GI) and insulin levels. Cereals are an important source of DF and the majority of DF is concentrated in the outer layers of grain, i.e., bran fraction. Chinese steamed bread (CSB) is a Chinese traditional fermented food, which has been widely consumed as a staple food in China. Hence, this research investigates how cereal bran inclusion in CSB effects the product from physical perspective and nutritional perspectives with reference to the glycaemic impact.

Methods: With respect to physical properties, specific volume, loaf height, moisture, and texture were measured by AACC methods. The nutritional quality of the bread was analysed by predicting glycaemic response using an in vitro digestion method.

Results: From physical properties of CSB, it can be seen that the specific volume reduced significantly (*p* < 0.05) as the addition of wheat bran increased. In textural properties of CSB, the addition of wheat bran increased the hardness, gumminess and chewiness but there was no significant difference revealed between 5% and 10% levels. This study also showed that addition of wheat bran can decrease the predicted glycaemic response of steamed bread by up to 39%.

Conclusions: This study illustrates the potential addition of wheat bran to improve the nutritional quality of CSB.

### 2.19. Effect of Rice Cooking Methods on Postprandial Glycaemic Response, Satiety and Palatability, and Chewed Particle Size Distribution

Lu, L.W., Venn, B.J. and Rush, E.

Background: Diets, which produce low glycaemic response, are relevant to prevention and management of obesity and diabetes. The aim was to investigate the effect of rice products and cooking-storing methods on postprandial blood glucose and the changes in satiety and palatability.

Methods: The randomised, cross-over experimental trial investigated the glycaemic responses, satiety and palatability (Visualised Analogue Scale (VAS)) scores of 28 healthy participants after consumed three rice samples (140 g ± 0.3 g), freshly cooked medium-grain-white, freshly cooked parboiled, and reheated parboiled (24-h storage at 4 °C and reheated to 65 °C), in each study visit. Postprandial blood glucose was recorded at 0, 15, 30, 45, 60, 90 and 120 min after rice consumption. Satiety (VAS score) was reported at 0, 30, 60, 90, and 120 min. Palatability (VAS score) was reported immediately after consumption. Glycaemic responses, satiety, and palatability among three rice samples were compared using repeated-measure-analysis of variance (ANOVA).

Results: The overnight cold-stored and reheated parboiled rice resulted in a significantly lower blood glucose concentration trajectory (42%, *p =* 0.01) than freshly cooked medium-grain white rice and 12% lower (*p =* 0.01) than freshly cooked parboiled rice. Longer chewing time (6.34 s/10 g) was observed in reheated parboiled rice compared with freshly cooked medium-grain white (*p =* 0.026) and higher palatability (visual appeal 2-fold higher (*p =* 0.001), smell 1-fold higher (*p =* 0.047), taste 1.5-fold higher (*p =* 0.018), and overall palatability 2-folder higher (*p =* 0.002)). No significant differences in satiety were observed (*p* > 0.050).

Conclusion: The effect of reheating on glycaemic response, chewing time and palatability shown in the present study may be considered a positive effect with regard to glycaemic regulation. Reheated parboiled rice replacing freshly cooked medium-grain white or parboiled rice in the habitual diet may reduce glycaemic overload in the daily diet.

### 2.20. Paying for the Price of Convenience—Comparing the Cost of Takeaway Meals with Their Healthy Home-Cooked Counterparts

Mackay, S., Xie, P., Vandevijvere, S., Lee, A. and Swinburn, B.

Background: Convenience and cost impact on people’s meal decisions with takeaways and pre-prepared food items often selected to save time and/or money. The cost of a set of popular takeaway meals was compared to similar but healthier home-made meals and home-assembled meals using pre-prepared ingredients in New Zealand.

Methods: The six most popular New Zealand takeaway meals were identified and compared to similar, but healthier, home-made and home-assembled meals. The meals prepared at home met criteria consistent with the New Zealand Eating and Activity Guidelines. The cost of each complete meal and the cost per 1 kg were calculated. The time-inclusive cost was calculated by adding the waiting or preparation time cost at the minimum wage.

Results: For five out of six popular meals, the mean costs of the home-made and home-assembled meals were cheaper than the mean cost of the takeaway meals. When the cost of time was added, for all six popular meals, the home-assembled meals were the cheapest, with either the home-made meal or takeaway meal the most expensive option. The meals prepared at home provided substantially less saturated fat and sodium and more vegetables than their takeaway counterparts. 

Conclusion: Home-made and home-assembled meals were healthier and cheaper options than takeaways when the cost of time was not included. Home-made meals took time to prepare so, when the cost of time was added, these became more expensive than most takeaway meals. Home-assembled meals were a quicker, more convenient option than home-made meals, but provided more sodium than the latter. 

### 2.21. Why Don’t They Just Grow Their Own Vegetables? The Role of Poverty in Nutrition in Aotearoa New Zealand

McKerchar, C.

Discourse analysis is a useful tool to explore how issues are framed. This presentation will explore how poverty and nutrition have been portrayed in the media through examples that usually state poverty is an outcome of individual or cultural failings rather than societal structures. The societal structures that lead to poverty and therefore poorer nutrition outcomes, will be discussed using the health inequalities theoretical framework by Williams. A public health ‘rights based’ approach to addressing food insecurity, that draws on international human rights frameworks will also be explored as a potential tool to reframe the issue of food insecurity in New Zealand.

### 2.22. What Is the Role of Kai and Nutrition in Manaakitanga? 

Matoe, L. 

To unfold the many layers to this question we must start with a Māori understanding of the creation of the universe for within this understanding we draw insights into the origins of food and the origins of eating the many foods we find in nature. This relational understanding of food allows us to understand the traits of the foods, their nutritional value and their spiritual value. This may appear rather abstract to many, but again, a Māori understanding of the world does not separate the physical from the spiritual. Rather, the Māori mind, as with many ancient cultures throughout the globe, is able to hold them both together in co-existence. When we consider our relationship with food through this worldview, what emerges is a Māori rationale for nutrition principles and eating wholefoods. From this rationale we move into the notion of Manaakitanga, a central function of Māori society from pre-European society and upheld in contemporary times. The function of manaakitanga is anchored by relationships whereby expressions of manaakitanga enhances relationships between groups and individuals, it shows how much we care and therefore the role of food in these expressions plays a very important part. In this opening presentation we will discuss the relational view of food and the role of food in expressions of manaakitanga from pre-European to contemporary Māori society to inform a deeper, broader understanding of the concept of manaakitanga and nutrition in Aotearoa.

### 2.23. Factors Associated with Nutrition Risk in Older Māori Living in the Bay of Plenty and Northland Regions of New Zealand

Maxted, E., Wham, C., Teh, R. and Kerse, N.

Background: Risk factors related to risk of malnutrition identified in other groups as have not been examined in Māori. The aim was to identify the prevalence of nutrition risk in older Māori and to identify cultural, social and physical factors associated with high nutrition risk. Methods: Māori aged 75 to 79 years living in the Northland and Bay of Plenty regions of New Zealand were assessed for nutrition risk using the validated screening tool ‘Seniors in the Community: Risk Evaluation for Eating and Nutrition’ (SCREENII). Demographic, physical and sociocultural data were collected.

Results: Of the 67 participants, two thirds (63%) were identified to be at high nutrition risk (SCREENII ≤ 50). More than half (56%) used te reo Māori language for everyday conversation and those who rated language and culture as moderately important to wellbeing were at lower nutrition risk. Controlling for other variables participants who reported that traditional foods were important and available to them had a higher waist to hip ratio and fewer depressive symptoms were at lower nutrition risk.

Conclusions: Cultural factors associated with nutrition risk are related to an indigenous view of health. Participants with a higher waist to hip ratio were at lower nutrition risk and this may be a protective factor for older Māori. Interventions to improve the nutrition status of older Māori need to be based on a holistic Māori worldview and acknowledge the importance of traditional Māori foods. 

### 2.24. Salty and Sweet—Where Is the Food Industry at with Improving the Foods Our Children Eat?

Monro, D. and Morley-John, J.

Background: The food our children commonly eat is often un-necessarily high in salt and sugar. This can develop life-long preferences for sweet and salty food and increase the risk of chronic diseases such as heart disease. Processed foods are major contributors of these nutrients; for example 75% of our salt comes from processed foods. The World Health Organisation has identified the food industry as part of the solution to addressing chronic diseases. 

Methods: Since 2007, the Heart Foundation has implemented a food reformulation programme focused on reducing salt (and more recently sugar) levels across processed foods. The programme is Ministry of Health funded, involves establishing voluntary targets and timeframes in partnership with the food industry for key food categories. Industry consultation is done on a one to one level and as an industry roundtable. Market analyses, modelling of potential impact, technical and commercial barriers are explored as part of setting category targets.

Results: Sodium targets have been set for thirteen categories. The objective of the programme is to have at least 80% of the market share (AC Nielson sales volumes) meeting the targets, which ensures high-volume foods in the category are prioritised. This objective has been met in the majority of key categories (e.g., bread, breakfast cereals, processed meats) resulting in 250 tonnes of salt per annum removed from targeted products. In 2016 sugar reduction targets have been set for breakfast cereal, tomato sauce and other foods frequently eaten by children. 

Conclusion: Continued focus on setting salt and sugar targets and product reformulation by companies to the targets has the potential to bring about population health improvement. Agreement from the key manufacturers on targets helps create a level playing field that they can all work towards, thereby minimising risk for companies. The targets also influence new product development. 

### 2.25. Combined Effect of Blackcurrant and Wholemeal Flours to Improve Health Promoting Properties and Reduce Predicted Glycaemic Response of a Model Food Cookie

Mofasser, H.A.K.M., Brennan, C.S., Brennan, M.A. and Mason, S.

Background: A diet with high glycaemic index, which causes rapid spikes in blood sugar level, can lead to disorders such as—significantly increased risk for type 2 diabetes, cardiovascular disease and obesity. These conditions are also linked to the progression of cognitive decline and neurodegenerative diseases including Alzheimer’s disease. The role of dietary fibre (DF) in disease prevention has been extensively investigated and prospective studies observed that increased DF intake decreased the risk of cardiovascular disease, reduced the risk of weight gain as well as the risk of type 2 diabetes, colon cancer and ameliorated brain and gut health. Blackcurrant powder (BC) is a rich source of dietary fibre and bioactive compounds among all berries. Wholemeal wheat, barley and oat flour contain high amount of fibre.

Methods: We have developed a model food cookie to investigate the glycaemic glucose equivalent (GGE) in vitro and antioxidant activities of three different wholemeal flours (wheat, barley and oat) with different replacement level (5%, 10% and 15%) of blackcurrant powder.

Results: Increasing the proportion of blackcurrant powder in the cookie resulted in a significant (*p* < 0.05) decrease in glucose release after in vitro digestion compared to the control (wheat control 624.4 mg/g, W15BC 440.0 mg/g; BCC-554.6 mg/g, B15BC-435.3 mg/g, OCC-523.6 mg/g, O15BC-415.9 mg/g at time 120 min). In addition, incorporation of blackcurrant powder in cookies up to 15% increased the antioxidant capacity. Several researchers have revealed that the bioactive compounds, especially polyphenols and anthocyanin of fruits have enzymes that have an inhibitory effect during the digestion process. 

Conclusion: Therefore, the combination of wholemeal flour and the bioactive compound rich blackcurrant can provide the potential to improve the nutritional value and reduce the glycaemic index of such foods.

### 2.26. How Do I Grow? A Health and Wellbeing Programme for Children in Early Childhood Care

Munday, K. and Wilson, M.

Background: In New Zealand, there is a high prevalence of childhood poverty and food insecurity which can impact on a family’s ability to provide high quality, nutrient dense foods for their children. The 2002 New Zealand National Nutrition Survey (Children) showed that only two out of five children ate the recommended daily servings of fruit and only three out of five ate the recommended daily servings of vegetables. To address the food insecurity issues faced by many of the children at a decile two kindergarten, staff decided to introduce a free lunch programme. In conjunction with this, a ten-week healthy-eating education programme was developed for the children and their families.

Methods: The education programme involved; structure bi-weekly education sessions, which introduced ‘healthy eating’ concepts through songs, stories and art; whanau cooking classes; tasting sessions; and the utilisation of the kindergarten’s vegetable patch to grow vegetables for process-cooking. Staff provided feedback regarding the implementation of the programme and the lunch service to highlight logistical challenges associated with its implementation.

Results: Changes in eating habits were primarily assessed through dietary questionnaires. The end-intervention 24-h dietary recall data showed a significant increase in calcium intake (*p* < 0.05) and the follow-up food frequency questionnaires indicated an increase in fruit and vegetables consumption. Follow-up feedback provided by parents and whanau showed that participation in the intervention had had a positive impact on the eating habits of whole families.

Conclusion: Whilst the lunch programme had a limited impact on the children’s nutrient intakes, the education programme was deemed a success by the parents and whanau who observed an increased willingness for the children to eat new fruits and vegetables and other foods following the intervention. The learnings from this intervention could help other pre-schools interested in setting up similar programmes.

### 2.27. Food Insecurity in New Zealand: Causes, Consequences and Cures

Parnell, W.R., Boston, G. and Simpson, J.

Food (and nutrition) security exists when all people at all times have physical and economic access to food which is safe and consumed in sufficient quantity to meet their dietary needs and food preferences and is supported by an environment of adequate sanitation, health services and care allowing for a healthy life. In New Zealand food insecurity is largely the result of lack of sufficient money for food although other socio-cultural factors exacerbate the condition. Unemployment, low levels of Government Benefits, low wages, accompanied by complex and less than ideal social conditions contribute to the causes of food insecurity.

National Nutrition Surveys have provided clear evidence that food insecurity at the household level is associated with poorer food choices, poorer nutrient intake levels and challenges to the maintenance of a healthy body weight particularly among women. Implicit within the condition of food insecurity is the concept of stress associated with obtaining food that is sufficient and available in a socially acceptable way. Thus food insecurity profoundly influences both physical and emotional health and affects the ability to participate fully in the life of the community.

Ultimately no remedy other than the provision of ‘sufficient’ money to a person or household to enable the purchase of sufficient appropriate food for health will counter food insecurity. Interventions proposed to alleviate food insecurity in NZ include: actions to release money for the purchase of household food by better distribution of money over all expenditures; enhancing food purchasing and preparation skills; household or community gardening projects; provision of affordable transport to food sources and better location of supermarkets; redistribution of and reduction of food otherwise wasted.

Evaluations of the effectiveness of such interventions are sparse. A qualitative Canadian study of rural food insecurity titled their discourse “When cooking skills, homegrown food and perseverance aren’t enough to feed a family”. Thus efforts focused on the economic underpinnings of food insecurity are of paramount importance.

### 2.28. Nutrition and Dysphagia Risk among Hospitalised Older Adults Admitted to the Assessment, Treatment and Rehabilitation Wards at Waitemata District Health Board hospitals

Patel, D., Richter, M., Allen, J. and Wham, C.

Background: Identifying those who are malnourished or at nutrition risk is essential for early nutrition intervention and improved health outcomes. Current data on the prevalence of nutrition risk in hospitalised older adults in NZ is limited. We therefore examined the prevalence of nutrition and dysphagia risk among adults aged 65–84 years newly admitted to AT&R wards in the Waitemata DHB.

Methods: An interviewer administered questionnaire was used to assess sociodemographic and health characteristics. Nutrition risk was assessed using the Mini Nutritional Assessment-Short Form (MNA-SF) and dysphagia risk using the 10-Item Eating Assessment Tool (EAT-10). Pearson Chi-Square tests were used to determine differences in dysphagia risk status and previous dietetic input between participants identified to be malnourished, at nutrition risk and with normal nutrition status using the MNA-SF

Results: A total of 89 assessments were completed in older adults (41 men), mean age 78.1 ± 5.9 years. Over two-thirds (70.8%) of participants were malnourished (27.0%) or at nutrition risk (43.8%) and a quarter (27.6%) were at risk of dysphagia. Compared to those with ‘at risk’ or with ‘normal’ nutrition status, malnourished participants were more likely to be at risk of dysphagia (54.5% vs. 15.4% & 23.1% respectively, *p =* 0.002); and were more likely to have had received previous dietetic input (50.0% vs. 35.9% & 15.4% respectively, *p =* 0.030). Key MNA-SF risk factor items for participants who were malnourished or at nutrition risk versus ‘normal’ nutrition status were severe reduced food intake within 3 months (45.8% & 7.7% vs. 0.0%); >3 kg unintentional weight loss within 3 months (62.5% & 30.8% versus 0.0%); and having reduced mobility (bed/chair bound) (20.8% & 10.3% vs. 0.0%) respectively.

Conclusion: A high proportion of hospitalised older adults recently admitted to the AT&R wards had compromised nutritional status. Routine screening is recommended to identify nutritional risk status and instigate appropriate nutritional care.

### 2.29. Nutrition and Dysphagia Risk among Newly Admitted Hospitalised Adults of Advanced Age 

Popman, A., Richter, M., Allen, J. and Wham, C.

Background: The 85+ age group is the fastest growing population segment in NZ. Prevalence of nutrition risk among community living octogenarians has been reported to range between 31%–49%, but is largely unknown in people of advanced age recently admitted to hospital Admission, Treatment and Rehabilitation (AT&R) wards. Aim: To establish the prevalence of nutrition risk among adults 85 years and older newly admitted to the AT&R wards at North Shore and Waitakere Hospitals in Auckland.

Methods: Participants were recruited within five days of admission to the AT&R wards. An interviewer administered questionnaire was used to assess sociodemographic and health characteristics, nutrition risk was assessed using the Mini Nutritional Assessment-Short Form (MNA-SF) and dysphagia risk assessed using the 10-item Eating Assessment Tool. Anthropometric measures were taken to assess body mass index (BMI), muscle mass (using bioimpedance scales) and grip strength (using a handgrip dynamometer). Pearson Chi-Square tests were used to examine differences in dysphagia risk between MNA-SF nutrition status groups. Pearson correlations were used to identify correlations between participant characteristics and nutrition status. 

Results: Assessments were completed in 88 advanced age adults (31 men), mean age 90.0 ± 3.7 years. As determined from the MNA-SF over two thirds (71.6%) of the participants were either malnourished (28.4%) or at high nutrition risk (43.2%). A third (29.5%) of the participants was at risk of dysphagia. Malnourished participants were more likely to be at risk of dysphagia (*p =* 0.015). The MNA-SF score positively correlated with BMI (*r* = 0.484, *p <* 0.001); grip strength in the dominant hand (*r* = 0.250, *p =* 0.026), and negatively correlated with dysphagia risk (*r* = −0.383, *p <* 0.001).

Conclusion: Malnutrition and high nutrition risk was prevalent among newly hospitalised adults of advanced age. Routine screening on admission is an important first step to identify those at nutrition risk. Dysphagia risk should be assessed in the malnourished to help shape appropriate intervention. 

### 2.30. What if Nutrition and Nutrients Could Treat Mental Illness? The Evidence to Date

Rucklidge, J. 

Despite the advent of medications and other therapies over the last 50 years, the rates of mental illness have been on the rise rather than a decline. Over the last decade, scientists have been uncovering an uncomfortable truth: What we eat is affecting our mental health. In this lecture, Prof Rucklidge will first describe what is known about dietary patterns and mental health and then discuss the recent paradigm shift of using broad based micronutrients to treat psychiatric disorders, reviewing the hypothesized mechanisms of action and the evidence to date. The talk intends to challenge our current treatment regime for mental disorders and suggest one alternative course of action.

### 2.31. Muriel Bell Lecture. Evolution Not Revolution: Nutrition and Obesity

Rush, E. 

Evolution may be defined as the “gradual development of something”. The word can be applied to a lifetime, to generations and to situations. I have been involved in health and education my entire working career, with an enthrallment with the amazing anatomy and physiology of the human body. The evolution of my understanding and experience stems from the training in physiological measurement at Green Lane Hospital; a world leader in cardiac and respiratory medicine in the late 60s and 70s. With family responsibilities teaching nurses and prenurses basic science, anatomy and physiology and applying in a practical way fed the desire to understand the body better. An MSc in physiology, exercise and immunity, followed by a PhD using stable isotopes as a measurement tool for body composition, energy expenditure, physical activity and an appreciation of the foods and nutrients people eat. An interest in ethnic differences in health and involvement in programmes and actions that will make a difference particularly for health of Māori, Pacific, Chinese and South Asian New Zealand people has continued with an appreciation of the importance of the developmental origins of health and disease. She is currently involved with Sport Waikato and Sport Northland in Project Energize (70,000 primary school children), Under 5 Energize (5000 pre-school children) and the Pacific Islands Families Study. Obesity is a poorly understood word and it is a form of malnutrition. The importance of translation of nutrition and physical activity health promotion research into practice in the real world and understanding HOW to do this; close the gap between efficacy and effectiveness in an equitable way is the current challenge. Real-life New Zealand examples will be shared.

### 2.32. Equipping Emerging Nutrition Leaders with Capacity to Work towards Food and Nutrition Security in Oceania 

Skeaff, S.A. on behalf of ONLP16

Background: Leadership and ability to communicate and collaborate are critical skills needed to build a strong nutrition workforce. The Oceanic Nutrition Leadership Program (ONLP) was developed with the vision and mission of “working together towards sustainable food and nutrition security for Oceania by developing, inspiring and connecting a new generation of visionary leaders in nutrition”.

Methods: Applicants (19 female, 3 male) from five countries across Oceania were selected by an independent committee for the inaugural ONLP course. Participants represented academia/research (50%), industry (9%), hospital/health service (14%), Government (23%) and non-government (4%) sectors. The program focused on leadership, communication, team-building, networking, policy, social responsibility and trans-disciplinary approaches to solving “wicked” problems. A post-program evaluation assessed participant satisfaction using a scale from 1 (dissatisfied) to 4 (completely satisfied).

Results: Of the 16 participants (73%) who completed the evaluation, the majority of participants reported they were completely satisfied (62%) with the course with no participants dissatisfied. Furthermore, the course met expectations for 75% of the cohort. A networking strategy, that employed social media, was established, and a declaration of intent were sanctioned. 

Conclusions: The ONLP was successful in establishing a strong network that has the capacity to positively influence food systems in Oceania.

### 2.33. The Effect of Regular Activity Breaks on Appetite: Secondary Analysis of the ABPA Study 

Smith, G., Haszard, J., Perry, T., Homer, A., Fenemor, S., Rehrer, N., Skeaff, C.M. and Peddie, M. 

Background: Sedentary behaviour is a risk factor for cardiovascular disease and adverse cardiometabolic responses. Taking regular breaks from sedentary time lowers post-prandial glycaemia; however, little is known about its effect on appetite.

Objective: To determine if appetite differs when taking regular activity breaks compared to prolonged sitting. 

Design: This trial was a randomised, cross-over, laboratory-based intervention. Thirty-six typically sedentary adults (mean age 25 year (SD 3.9), BMI 23.9 (SD 3.9)) completed four two-day interventions (6.5 h on Day 1, 5 h on Day 2), each separated with ~5 days washout: Uninterrupted sitting on both days (SED); sitting interrupted with 2-min walks every thirty minutes (regular activity breaks (RAB)); sitting combined with a 30 min walk at the end of Day 1 (SED + PA); and regular activity breaks (on both days) combined with a 30 min walk at the end of Day 1 (RAB + PA). During each two-day intervention, participants completed a five-question, visual analogue scale appetite and satiety questionnaire every thirty minutes. The responses were combined into a single appetite score that measured overall hunger. Area under the curve (AUC) for overall hunger was calculated from directly following breakfast for ~3 h. 

The thirty minutes of physical activity at the end of Day 1 (SED + PA and RAB + PA) had no influence on appetite scores; therefore, the SED and SED + PA was combined to a single “SED” intervention, and the RAB and RAB + PA to a single “RAB” intervention. 

Outcomes: There was no significant difference in appetite AUC between the RAB and the SED interventions (Day 1: standardised effect 0.08; 95% CI −0.27, 0.11; *p =* 0.394. Day 2: standardised effect 0.05, 95% CI −0.13, 0.23; *p =* 0.588).

Conclusion: Interrupting prolonged sitting with regular activity breaks does not appear to increase appetite. Therefore, longer-term investigation into the effects of regular activity breaks on energy balance may be warranted. 

### 2.34. The Effects of Sensory Issues on Mealtime Behaviours and Food and Nutrient Intake in Children with Autism Spectrum Disorder

Taylor, N., Mazahery, H., Conlon, C.A., Beck, K., Mugridge, O.A.R. and von Hurst, P.R.

Background: Sensory issues are highly prevalent in children with Autism Spectrum Disorder (ASD) and have been associated with difficult mealtime behaviours. It is not known if sensory issues are associated with food or nutrient intake in ASD children living in New Zealand (NZ). Nutritional deficits during development could have compounding effects on cognition and behaviour in ASD.

Methods: In this cross-sectional observational study we investigated difficult mealtime behaviour and food and nutrient intakes associated with the sensory issues of 113 children aged 2.5–8 years with ASD in NZ. The Sensory Processing Measure (SPM), Behavioural Paediatric Feeding Assessment Scale, Dietary Intake for Child’s Eating (DICE), and four-day food diaries were used to measure sensory issues, difficult mealtime behaviours, food intake, and nutrient intake, respectively. Weight and height were also measured to determine body mass index percentile.

Results: Overall, 90.2% of children had sensory issues. An increase in sensory issues corresponded to an increase in difficult mealtime behaviours (*r* = 0.264, *p =* 0.007). Social participation issues were inversely associated with the total DICE score (*r* = −0.305, *p =* 0.003). Approximately 30% of children were overweight or obese. More than 50% of the children did not meet Ministry of Health (MoH) recommendations for servings of fruit, vegetables, breads and cereals, milk and milk products, or nutrient intakes for folate, vitamin C, fibre, and iron.

Conclusion: Sensory issues are highly prevalent in ASD children and are associated with difficult mealtime behaviours. The majority of children were not meeting MoH guidelines, however sensory issues did not appear to be associated with food and nutrient intakes. Nutritional intervention is required in a number of children with ASD to treat or prevent overweight/obesity and to ensure food and nutrient intake recommendations are met.

### 2.35. Estimating Free Sugars Intake in New Zealand

Te Morenga, L., Kibblewhite, R., Nettleton, A. and McLean, R.

Background: The World Health Organisation (WHO) recommends free sugars intakes should be less than 10% of total energy intake (TE) and ideally less than 5% TE. Free sugars are defined as sugars added to foods and beverages by the manufacturer, cook, or consumer; and includes sugars naturally present in honey, syrups, fruit juices and concentrates. Monitoring free sugars intakes is difficult in New Zealand as our Food Composition Database (NZFCD) does not provide nutrient information for free sugars. 

Methods: We developed free sugars estimates for all foods in the 2006 and 2010 NZFCD using an adaption of a previously published 10-step protocol. We then estimated free sugars intakes of NZ adults using dietary intake data from the NZ Adult Nutrition Survey 2008/09. Survey weighted estimates were calculated by age class, sex, and ethnicity with adjustment for intraindividual variation; prioritised ethnicity was used.

Results: Free sugars content (g/100 g) for 2779 relevant foods and beverages in the NZFCD were estimated. The median intake of free sugars in all adults was 57 g/day which equated to 11.1% TE. Intakes were highest among younger age groups; younger males aged 15–18 and 19–30 years had median consumption levels of 84 g/d (13% TE) and 75 g/d (12% TE) respectively while younger females (15–30 years) consumed more than 14% TE from free sugars. Overall 58% of the total population consumed more that 10% TE and 91% consumed more than 5% TE from free sugars. 

Conclusion: Free sugars intakes of the majority of NZ adults are higher than desirable, especially amongst younger adults. 

### 2.36. Nutritional Adequacy and 24-Month Fracture Occurrence in Māori and Non-Māori of Advanced Age: LiLACS NZ

Towgood, A., Wham, C., Teh, R., Moyes, S., Rolleston, A. and Kerse, N.

Background: The role of nutrition in fracture occurrence among octogenarians is unclear. The aim was assess the dietary intake of micronutrients necessary for bone health among Māori and non-Māori aged 80–90 years and fracture occurrence 24 months post dietary assessment. 

Methods: Nutritional assessments were completed by 574 Māori and non-Māori using a validated repeat 24 h MPR protocol. Fracture occurrence was measured over a 24 month period following the 2 × 24 h MPR and included self-reported and hospitalised fracture occurrences. Demographic, lifestyle and deprivation data were collected.

Results: A quarter (23.7%) of the participants sustained a fracture over a 24 month period (22.5% Māori women, 13.7% Māori men and 23.6% non-Māori women, 17.3% non-Māori men). Compared to those not fractured, non-Māori women who fractured were 1.1 times more likely to be financially insecure (*p =* 0.03) and among Māori women who fractured, inability to afford to eat properly was 3.3 times more likely (*p =* 0.01). Regardless of fracture status median intakes of calcium, vitamin D and magnesium were low. For men and women respectively, calcium intakes were 559 mg vs. 539 mg for Māori; 748 mg vs. 672 mg for non-Māori compared to the EAR of 1100 mg. Vitamin D intake from food was <4 µg for all participants compared to the EAR of 15 µg/day for men and women. Magnesium intakes were 259 mg vs. 204 mg for Māori and 271 mg vs. 238 mg for non-Māori men and women respectively; compared to the EAR for men 350 mg and women 265 mg. Fractured compared to non-fractured Māori women consumed less vitamin D (2.0 µg vs. 3.0 µg) (*p =* 0.01) and magnesium (143.0 mg vs. 211 mg) (*p =* 0.03) from food.

Conclusion: Promoting increased intakes of affordable and culturally acceptable foods may be advantageous to increase calcium and magnesium intakes. Although vitamin D intakes were minimal from food; participants may have received supplementary vitamin D and further investigation is warranted.

### 2.37. Mineral Content of Mixed Vegetable Juice Produced Using Two Different Domestic Juicers

Vanhanen, L.P. and Savage, G.P. 

Background: Home juicing using readily available juicing machines is now commonplace. It is promoted not only as a mechanism to quench your thirst and provide refreshment, but also as a source of extra nutrients, vitamins and minerals. Vegetable juicers can be classified into two basic types; a masticating juicer (MJ), separates out the fibrous portion (pulp) of the juice, which is then discarded and a high speed blender (HSB), which retains the fibrous fraction of the juice. 

Methods: A vegetable juice recipe consisting of spinach, apple, celery, cucumber, green pepper, red capsicum, lemon and parsley was juiced using both juicers. A further recipe was prepared with the same proportions of ingredients except that the spinach content was doubled. 

Results: Fifteen different minerals were identified in measurable amounts in the juice and the discarded fibre fraction, with levels ranging from 0.001 to 359.36 mg/100 g juice FW, for Cd and K respectively. Juices prepared from mixtures containing high levels of spinach contained measurably higher amounts of all minerals except calcium. Analysis showed that these juices contained high levels of oxalates bound to calcium and therefore would be unlikely to be absorbed in the digestive tract when consumed. 

Conclusions: Juices prepared using a MJ juicer always contain lower levels of minerals compared to juices prepared from a HSB juicer. The lower levels of minerals in the MJ juicer contain significantly lower levels of minerals are they are discarded in the fibre fraction.

### 2.38. A Randomised cross over Trial to Examine the Effect of Kiwifruit on Protein Digestion in Healthy Adult Males 

Wallace, A., Sarah Eady, S., Drummond, D., Hedderley, D., Juliet Ansell, J. and Gearry, R. 

Background: ‘Hayward’ kiwifruit anecdotally are associated with improved gastrointestinal comfort following consumption of high protein meals possibly because of the presence of a protease enzyme ‘actinidin’. The study aimed to use Smartpill™ technology to investigate the acute effect of kiwifruit with actinidin (*Actinidia chinensis* var. *deliciosa* ‘Hayward’) and kiwifruit without actinidin (*A. chinensis* var. *chinensis* ‘Hort16A’) on digestion of a large protein meal. 

Methods: Ten healthy male subjects were recruited. Participants attended the clinic three times, having fasted overnight. They consumed a test meal consisting of 400 g lean steak and two ‘Hort16A’ or two ‘Hayward” kiwifruit. Subjects completed visual analogue scales (VAS) rating feelings of hunger, satisfaction, fullness and comfort, and swallowed a SmartPill before completing further VAS scales. After 5 h participants consumed an *ad libitum* lunch to assess satiety. SmartPill transponders were worn for five days. 

Results: There were no significant differences in gastric emptying time, small bowel or colonic transit time between the two kiwifruit arms of the study measured by Smartpill. Similarly, no significant differences were observed in VAS satiety measures or energy consumption at the *ad libitum* meal. However, measurement of overall gastric comfort tended to be lower, and bloating was significantly reduced following consumption of the steak meal with ‘Hayward’ kiwifruit (*p* < 0.028). 

Conclusions: The SmartPill is marketed as a diagnostic tool for patients presenting with gastrointestinal disorders and is usually used with a standard “SmartBar”. This small pilot study suggests that it is less likely to measure gastric emptying effectively following a high protein meal, as it may be delayed due to the meals’ physical consistency. However, green kiwifruit, containing actinidin, may reduce bloating and other measures of gastric discomfort in healthy males. Possible future studies could use repeated measures with more readily digested protein and larger numbers of participants.

### 2.39. Exploring the Perceived Relationship between Chronic Stress and Eating Behavior—A Qualitative Study 

Walker, E. and Polak, M.A.

Background: There is limited qualitative research surrounding the perceived relationship between stress and food choices. Stress has been associated with changes in eating behaviours. Previous research established a link between acute stress and a decrease in dietary intake, however, there is not enough evidence to determine the effect of chronic stress. This study explored Canterbury residents’ perceptions of the relationship between chronic stress and behaviours and possible reasoning as to why this relationship presents. 

Methods: We recruited Christchurch residents who were present for the two large the Canterbury earthquakes (September 2010 and February 2011). Six individuals participated in this study and underwent a semi-structured interview. The combination of questions was unique to this study and the questions based on perceived dietary behaviours, stress, earthquake experiences and the relationship between chronic stress and eating behaviours. Data were content analysed, looking for common themes and then member checked. 

Results: Common themes included an overall decrease in appetite among individuals. Furthermore, the types of food being chosen were substituted to energy dense nutrient void foods whilst healthier alternatives, such as fruits and vegetables, decreased. In addition, participants perceived that poor food choice may be used as a coping mechanism for chronic stress and consuming these types of foods provide enjoyment, a sense of control, and convenience. 

Conclusions: The study successfully explored individuals’ perceptions of the relationship between stress and eating behaviours. By understanding how factors such as chronic stress affect dietary behaviours, nutritionists, dieticians, and other health professionals will be able to appreciate and understand client perceptions, and thus work more effectively to promote health and well-being. 

### 2.40. Screening for Nutrition Risk and Dysphagia among Older Adults Recently Admitted to Age Related Residential Care within the Waitemata DHB Region

Watkin, R.S., Gammon, C.S., Allen, J. and Wham, C.

Background: The prevalence of nutrition risk among community living older people in New Zealand has been reported to range between 31%–49%, but is largely unknown in age related residential care (ARRC). This study aimed to investigate nutrition risk and dysphagia in older adults recently admitted to an ARRC facility (rest home and hospital level care) in the Waitemata District Health Board (DHB) region. 

Methods: An interviewer-administered questionnaire was used to assess sociodemographic and health characteristics. Nutrition risk was assessed using the Mini Nutritional Assessment-Short Form (MNA-SF) and dysphagia risk using the 10-item Eating Assessment Tool (EAT-10). Descriptive analyses were used to describe the group. 

Results: Assessments were completed for 53 older adults (30 female, 23 male), mean age 88 ± 6.7 year, with 70% aged ≥85 year. The majority (83%) were of New Zealand European ethnicity, 30 were in rest home and 23 in hospital care. Polypharmacy was prevalent (79% taking ≥5 medications regularly). Over 90% of participants were either malnourished (47%) or at risk of malnutrition (43%) and 32% were at risk of dysphagia. Malnourished participants were more likely to report weight loss (>3 kg) than those at risk of malnutrition (56% vs. 13% respectively), less likely to go out (16% vs. 57%) and were more likely to be at risk of dysphagia (52% vs. 17%). The prevalence of malnutrition and dysphagia risk was higher amongst participants in hospital care compared to rest home care.

Conclusions: The majority of older adults recently admitted to ARRC facilities were either malnourished or at high nutrition risk. This highlights the necessity of routine nutrition screening on admission. Positive screening should lead to immediate dietetic referral so a clear nutritional care pathway can be planned. Further investigation to determine the factors that contribute to nutrition risk in ARRC facilities is warranted.

### 2.41. Meeting the Diverse Nutritional Needs of Older Adults

Wham, C. 

Background: Older adults are a heterogeneous group and have unique nutrient needs. In NZ National Nutrition Survey data are aggregated over 70 years for non-Māori and over age 55 years for Māori. Older adults have different dietary requirements to younger people however the nutritional needs of people over 80 years are largely unknown. The aim was to overview findings on the nutritional status of those over 80 years. 

Methods: As part of the longitudinal cohort study LILACS NZ recruited 937 (421 Māori; 526 non-Māori) octogenarians. Nutritional assessments were undertaken at baseline using the ‘Seniors in the Community: Risk Evaluation for Eating and Nutrition ‘(SCREEN II)’and at follow up using the 2 × 24 h MPR method.

Results: Half (49%) of Māori and 38% of non-Māori participants were at high nutrition risk (SCREENII score < 49). For Māori participants independent risk factors were younger age, lower education, living alone and depressive symptoms. For non-Māori high nutrition risk was associated with female gender, living alone, a lower physical health related quality of life and depressive symptoms. At 12 months follow up median energy intake for Māori, was 1779 kcal/day for men and 1433 kcal/day for women with 16.3% energy derived from protein, 43.3% from carbohydrate and 38.5% from fat. Median energy intake was 1887 kcal/day and 1497 kcal/day for non-Māori men and women respectively with 15.4% of energy derived from protein, 45% from carbohydrate and 36.7% from fat. More than half of the Māori and non-Māori participants had intakes below the EAR for calcium, magnesium, selenium, vitamin B_6_ (Māori women only), folate (women only) and zinc (men only). The AI for vitamin E was not met by more than half of Māori women and all men.

Conclusions: These unique cross sectional data address an important gap in our understanding of nutritional status of advanced age adults and highlight a lack of age appropriate NRVs. Assessment of the impact of food and dietary intake on functional indices that affect quality of life in older adults is needed to determine age appropriate dietary requirements.

### 2.42. Effects of a Healthier Snack on Glycaemia, Satiety, and Habitual Snacking Behaviour

Yan, M. and Rush, E.

Background: There is evidence that government-led food reformulation initiatives improve the quality of food, e.g., to reduce salt intake. However, most actions have involved voluntary commitments from industry. There is also a call for high value nutrition products but the focus is on export and sales rather than improvement in public health. 

Methods: Between 2012 and 2015, in partnership with a food manufacturer, an eight ingredient snack bar branded Nothing Else has been developed, which has a good nutrient profile and a low glycaemic index (52). It is now in commercial production. The prototype was tested against two top-selling snack bars for the acute glycaemic and satiety responses at serving size portions. Both top-selling snack bars had a poor nutrient profile. In a stepped-wedge 6-week intervention, effects of daily consumption of the snack bar on snacking habits and glycated haemoglobin (HbA1c) were investigated.

Results: The investigation on glycaemia and satiety (*n =* 26) demonstrated 30% lower blood glucose area-under-the-curve over 2-h for the Nothing Else bar compared with one of the commercial bars (*p <* 0.001) of equal weight. At 45 min after eating, the Nothing Else bar induced the highest fullness rating and lowest hunger rating. Consumption of the Nothing Else bar as the main snack choice for 6 weeks resulted in a significant reduction in intake of biscuits, cakes and pies (~2 servings/week, *p* ˂ 0.05). Twenty participants (71.4%) out of 28 experienced a decrease or no change in HbA1c (range 0–4 mmol/mol), and for 8 participants, HbA1c increased (range 0.5–2.5 mmol/mol).

Conclusions: The Nothing Else bar, with its nutrient profile, is a healthier option for improvement of glycaemia, satiety in the short term and in the longer term, habitual snacking behavior and HbA1c: one small step towards providing a food environment that is supportive of a healthier diet.

## 3. Affiliations

- Aggio, R., Nutrition and Dietetics, University of Auckland, Auckland, New Zealand; University of Liverpool, Liverpool, United Kingdom
- Allen, J., Waitemata District Health Board, Auckland, New Zealand
- Ansell, J., Zespri International Ltd., Mount Maunganui, New Zealand
- Ansell, S., School of Food and Nutrition, Massey University, Auckland, New Zealand
- Bell, T., The Ferrier Institute, Victoria University of Wellington, Petone, New Zealand
- Beck, K.L., School of Food and Nutrition, Massey University, Auckland, New Zealand
- Borich, A., Department of Human Nutrition, University of Otago, Dunedin, New Zealand
- Boston, G., Medical School, University of Otago, Dunedin, New Zealand
- Breier, B.H., School of Food and Nutrition, Massey University, Auckland, New Zealand
- Brennan, C.S., Centre for Food Research and Innovation, Department of Wine, Food and Molecular Biosciences, Lincoln University, Lincoln, New Zealand
- Brennan, M.A., Centre for Food Research and Innovation, Department of Wine, Food and Molecular Biosciences, Lincoln University, Lincoln, New Zealand
- Brown, R., Department of Human Nutrition, University of Otago, Dunedin, New Zealand
- Buch, T., School of Population Health, University of Auckland, Auckland, New Zealand; School of Food and Nutrition, Massey University, Auckland, New Zealand
- Butts, C., New Zealand Institute for Plant and Food Research Ltd., Palmerston North, New Zealand
- Cairncross, C.T., AUT University, Auckland, New Zealand
- Camargo Jnr, C.A., Massachusetts General Hospital, Boston, MA, USA
- Coad, J., School of Food and Nutrition, Massey University, Palmerston North, New Zealand
- Conlon, C.A., School of Food and Nutrition, Massey University, Auckland, New Zealand
- Cooke, R., Department of Human Nutrition, University of Otago, Dunedin, New Zealand
- Desai, A.S., Centre for Food Research and Innovation, Department of Wine, Food and Molecular Biosciences, Lincoln University, Lincoln, New Zealand
- Dinnan, H., New Zealand Institute for Plant and Food Research Ltd., Palmerston North, New Zealand
- Drummond, L., Drummond Food Science Advisory Ltd., Akaroa, New Zealand
- Eady, S., The New Zealand Institute for Plant and Food Research Limited, Lincoln, Christchurch, New Zealand
- Ellett, S., Nutrition and Dietetics, University of Auckland, Auckland, New Zealand; Nutrigenomics NZ, Auckland, New Zealand
- Elmslie, J., Department of Psychological Medicine University of Otago, Christchurch and Specialist Mental Health Service, Canterbury District Health Board (CDHB), Christchurch, New Zealand
- Eyres, L., ECG Consulting, Auckland, New Zealand
- Fenemor, S., School of Physical Education, Sport & Exercise Sciences, University of Otago, Dunedin, New Zealand
- Ferguson, I.B., Ministry for Primary Industries, Auckland, New Zealand
- Ferguson, L.R., Nutrition and Dietetics, University of Auckland; Nutrigenomics NZ, Auckland, New Zealand
- Funaki-Tahifote, M., Pacific Heartbeat, National Heart Foundation, Auckland, New Zealand
- Gammon, C.S., School of Food and Nutrition, Massey University, Auckland, New Zealand
- Gearry, R., Department of Medicine, University of Otago, Christchurch, New Zealand
- Golding, M., School of Food and Nutrition, MIFST, Massey University, Auckland, New Zealand
- Grant, C.C., University of Auckland, Auckland, New Zealand
- Greig, F., Network Communication, Auckland, New Zealand
- Hashimoto, S., Massey Institute of Food Science and Technology, Massey University, Albany, New Zealand; Heart Foundation of New Zealand, Auckland, New Zealand
- Haszard, J.J., Department of Human Nutrition, University of Otago, Dunedin, New Zealand
- Hedderley, D., The New Zealand Institute for Plant and Food Research Limited, Palmerston North, New Zealand
- Henderson, L., School of Food and Nutrition MIFST, Massey University, Auckland, New Zealand
- Herath, T., New Zealand Institute for Plant and Food Research Ltd., Palmerston North, New Zealand
- Higginson, C.A., School of Human Nutrition, Massey University, Auckland, New Zealand
- Hofmann, R.W., Food Group, Agriculture and Life Sciences, Lincoln University, Lincoln, New Zealand
- Homer, A., Department of Human Nutrition, University of Otago, Dunedin, New Zealand
- Houghton, L.A., Department of Human Nutrition, University of Otago, Dunedin, New Zealand
- Ichhpuniani, B., School of Food and Nutrition, Massey University, Auckland, New Zealand
- Jayasinghe, S.N., School of Food and Nutrition, MIFST, Massey University, Auckland, New Zealand
- Jesuthasan, A., Nutrition and Dietetics, University of Auckland, New Zealand; Nutrigenomics NZ, Auckland, New Zealand
- Jones, L., School of Physical Education, Sport and Exercise Sciences, University of Otago, Dunedin, New Zealand
- Kenny, S., McDonald’s Restaurants (NZ) Ltd., Auckland, New Zealand
- Kerse, N., Department of General Practice and Primary Health Care, University of Auckland, Auckland, New Zealand
- Kibblewhite, R., Department of Human Nutrition, University of Otago, Dunedin, New Zealand
- Kindleysides, S., School of Food and Nutrition MIFST, Massey University, Auckland, New Zealand
- King, B., Pegasus Health, Christchurch, New Zealand
- Klunklin, W., Food Group, Agriculture and Life Sciences, Lincoln University, Lincoln, New Zealand
- Kruger, R., School of Food and Nutrition, Massey University, Auckland, New Zealand
- Laing, B., Nutrition and Dietetics, University of Auckland, Nutrigenomics NZ, Auckland, New Zealand
- Lee, A., Queensland University of Technology, Brisbane, Griffith University, Curtin University, Australia
- Liu, W., Department of Wine, Food and Molecular Biosciences, Faculty of Agriculture and Life Sciences, Lincoln University, Christchurch, New Zealand
- Lock, D., Institute of Food Research, Norwich, United Kingdom
- Lu, L.W., School of Sport and Recreation, Faculty of Health and Environmental Sciences, Auckland University of Technology, Auckland, New Zealand
- Mackay, S., School of Population Health, University of Auckland, Auckland, New Zealand
- McDonald, B., School of Food and Nutrition, Massey University, Auckland, New Zealand
- McKerchar, C., Department of Population Health, University of Otago, Christchurch, New Zealand
- McLean, R., Department of Human Nutrition, University of Otago, Dunedin, New Zealand; Edgar Diabetes and Obesity Research Centre, University of Otago, Dunedin, New Zealand; Department of Preventive and Social Medicine, University of Otago, Dunedin, New Zealand
- McNaughton, S.A., Institute for Physical Activity and Nutrition, School of Exercise and Nutritional Sciences, Deakin University, Melbourne, Australia
- Marlow, G., Nutrition and Dietetics, University of Auckland, Nutrigenomics NZ, Auckland, New Zealand
- Martell, S., New Zealand Institute for Plant and Food Research Ltd., Palmerston North, New Zealand
- Mason, S., Department of Wine, Food and Molecular Biosciences, Lincoln University, Lincoln, New Zealand
- Matoe, L., Kii Taki Ltd. and Te Kaahui o Rauru, Taranaki, Waverly, New Zealand
- Maxted , E., Dietetics, Northland District Health Board, Whangarei, New Zealand
- Mazahery, H., School of Food and Nutrition, Massey University, Auckland, New Zealand
- Monro, D., The National Heart Foundation of New Zealand, Auckland, New Zealand
- Morgan, S., COMET Auckland, Auckland, New Zealand
- Morely-John, J., The National Heart Foundation of New Zealand, Auckland, New Zealand
- Mofasser H.A.K.M., Department of Wine, Food and Molecular Biosciences, Lincoln University, Lincoln, New Zealand
- Moyes, S., General Practice and Primary Health Care, University of Auckland, Auckland, New Zealand
- Mugridge, O., School of Food and Nutrition, Massey University, Auckland, New Zealand
- Munday, K., Eastern Institute of Technology, Taradale, Napier, New Zealand
- Nettleton, A., Department of Human Nutrition, University of Otago, Dunedin, New Zealand
- Parnell, W., Department of Human Nutrition, University of Otago, Dunedin, Otago, New Zealand
- Patel, D., School of Food and Nutrition, Massey University, Auckland, New Zealand
- Paturi, G., Plant and Food Research, Auckland, New Zealand
- Peddie, M., Department of Human Nutrition, University of Otago, Dunedin, New Zealand
- Perry, T., Department of Human Nutrition, University of Otago, Dunedin, New Zealand
- Polak, M.A., Department of Applied Science and Allied Health, Ara Institute of Canterbury, Christchurch, New Zealand
- Popman, A., School of Food and Nutrition, Massey University, Auckland, New Zealand
- Rehrer, N., School of Physical Education, Sport & Exercise Sciences, University of Otago, Dunedin, New Zealand
- Richter, M., School of Food and Nutrition, Massey University, Auckland, New Zealand
- Rolleston, A., Department of General Practice and Primary Health Care, University of Auckland, Auckland, New Zealand
- Rucklidge, J., Clinical Psychology, University of Canterbury, Christchurch, New Zealand
- Rush, E., Child Health Research Centre, Auckland University of Technology, Auckland, New Zealand; School of Sport and Recreation, Faculty of Health and Environmental Sciences, Auckland University of Technology, Auckland, New Zealand; AUT Food Network, Auckland University of Technology, Auckland, New Zealand
- Savage, G., Food Science, Faculty of Agriculture and Life Sciences, Lincoln University, Lincoln, New Zealand
- Schramm, C., Network Communication, Auckland, New Zealand
- Schrijvers, J., School of Food and Nutrition, Massey University, Auckland, New Zealand
- Serventi, L., Department of Wine, Food and Molecular Biosciences, Faculty of Agriculture and Life Sciences, Lincoln University, Christchurch, New Zealand
- Sims, I., The Ferrier Institute, Victoria University of Wellington, Petone, New Zealand
- Simpson, J., Department of Women’s and Children’s Health, University of Otago, Dunedin, New Zealand
- Skeaff, S.A., Department of Human Nutrition, University of Otago, Dunedin, New Zealand
- Skidmore^,^ P.M.L., Department of Human Nutrition, University of Otago, Dunedin, New Zealand
- Smith, G., Department of Human Nutrition, University of Otago, Dunedin, New Zealand
- Stewart, N., Conifer Grove School, Auckland, New Zealand
- Stonehouse, W., Food and Nutrition Flagship, Commonwealth Scientific and Industrial Research Organisation (CSIRO), Adelaide, South Australia, Australia
- Swinburn, B., World Health Organization Collaborating Centre for Obesity Prevention, Deakin University, Australia; School of Population Health, University of Auckland, Auckland, New Zealand
- Taylor, N., School of Food and Nutrition, Massey University, Auckland, New Zealand
- Taylor, R.W., Department of Medicine, University of Otago, Dunedin, New Zealand; Edgar Diabetes and Obesity Research Centre, University of Otago, Dunedin, New Zealand
- Teh, R., Department of General Practice and Primary Health Care, University of Auckland, Auckland, New Zealand
- Te Morenga, L., Department of Human Nutrition, University of Otago, Dunedin, New Zealand; Riddet Institute, New Zealand; Edgar Diabetes and Obesity Research Centre, University of Otago, Dunedin, New Zealand
- Towgood, A., School of Food & Nutrition, Massey University, Auckland, New Zealand
- Vandevijvere, S., School of Population Health, University of Auckland, Auckland, New Zealand
- Vanhanen, L., Food Science, Faculty of Agriculture and Life Sciences, Lincoln University, Lincoln, New Zealand
- Venn, B., Department of Human Nutrition, University of Otago, Dunedin, New Zealand
- von Hurst, P.R., School of Food and Nutrition, Massey University, Auckland, New Zealand
- Walia, N., School of Food and Nutrition, Massey University, Auckland, New Zealand
- Walker, E., Department of Applied Science and Allied Health, Ara Institute of Canterbury, Christchurch, New Zealand
- Wallace, A., The New Zealand Institute for Plant and Food Research Limited, Lincoln, Christchurch, New Zealand
- Walsh, D.C.I., Institute for Natural and Mathematical Sciences, Massey University, Auckland, New Zealand
- Watkin, R.S., School of Food and Nutrition, Massey University, Auckland, New Zealand
- Wham, C., School of Food and Nutrition, Massey University, Auckland, New Zealand
- Wilson, M., Independent Nutrition Consultant, Palmerston North, New Zealand
- Xie, P., Faculty of Medical and Health Sciences, University of Auckland, Auckland, New Zealand
- Yan, M., AUT Food Network, Auckland University of Technology, Auckland, New Zealand; Unitec Institute of Technology, Auckland, New Zealand

