# Correction: Rachel Brown and Sheila Skeaff. Nutrition Society of New Zealand Annual Conference Held in Christchurch, New Zealand, 8–9 December 2016. *Nutrients* 2017, *9*, 348

**DOI:** 10.3390/nu9060618

**Published:** 2017-06-16

**Authors:** Rachel Brown, Sheila Skeaff

**Affiliations:** Department of Human Nutrition, University of Otago, Dunedin 9054, New Zealand; sheila.skeaff@otago.ac.nz

We would like to submit the following as a correction to our recently published Special Issue on the annual conference and scientific meeting of the Nutrition Society of New Zealand, 2016 [1]. The following abstract was inadvertently omitted from the proceedings.

2.43. Food ‘Costs’

Mainvil, L.

Background: Economic (structural) factors, including income and food price, may partially explain socioeconomic inequalities in diet quality, obesity and health outcomes. Internationally, healthy foods and dietary patterns tend to cost more than less healthy options. Calories from energy-dense foods (refined grains, fats/oils, added sugars) are relatively low cost; whereas, calories from nutrient-rich foods (fresh fruits/vegetables, lean meats/chicken) are relatively high cost. Greater variety and cultural acceptability also increases food costs. The NZ Food Cost Survey has been monitoring retail food prices for a weekly basket of healthy food in New Zealand since 1972. In 2014 methodological updates ensured food types and amounts were culturally acceptable and achieved both dietary guidelines and nutrient requirements.

Methods: The availability and retail price (ignoring ‘specials’) of 161 foods in four large supermarkets in Auckland, Wellington, Christchurch and Dunedin were recorded annually. The weekly estimated food costs for individuals following a basic (cooked from scratch), moderate and liberal diet were calculated by city.

Results: Auckland ‘basic’ healthy food costs ranged from $27 (1 year old) to $67 (adolescent male) per week in 2016. For example, an Auckland household of four, basic healthy food costs were $233 per week (man $64, woman $55, adolescent boy $67, 10 years old $47), which is 41% of a full-time (pre-tax) income on the minimum wage. Most of this cost came from fruits/vegetables (30%), meats/proteins (27%) and dairy (17%). While food prices in New Zealand fell slightly in 2016, food costs have been rising over time. Full results are reported elsewhere (http://www.otago.ac.nz/humannutrition/research/food-cost-survey).

Conclusions: Threats to healthy food affordability include inadequate incomes (rising unemployment, declining real wages/benefits due to rising housing and other costs), reduced food supply (global climate change impacts), and increased food demand (global food security, population growth, bio-fuels). These threats can be managed with sustainable environmental, agricultural/food chain, economic and social welfare policies.

**Affiliation:**

Mainvil, L., Department of Human Nutrition, University of Otago, Dunedin, New Zealand.
